# Rare‐Earth–Functionalized Carbon Dot Hydrogels as Mechanically Robust Antibacterial Wound Dressings

**DOI:** 10.1002/adhm.71608

**Published:** 2026-08-17

**Authors:** Shih‐Han Tsai, Ramadhass Keerthika Devi, Muthusankar Ganesan, Guo‐Ping Chang Chien, Chun Che Lin

**Affiliations:** ^1^ Institute of Organic and Polymeric Materials Research and Development Center For Smart Textile Technology National Taipei University of Technology Taipei Taiwan; ^2^ Super Micro Mass Research and Technology Center Cheng Shiu University Kaohsiung Taiwan; ^3^ Center For Environmental Toxin and Emerging‐Contaminant Research Cheng Shiu University Kaohsiung Taiwan; ^4^ Institute of Environmental Toxicology and Emerging Contaminants Cheng Shiu University Kaohsiung Taiwan

**Keywords:** antibacterial, anti‐inflammatory, biocompatibility, dipotassium glycyrrhizate, hydrogels, lanthanum‐functionalized carbon dots, wound dressing

## Abstract

Hydrogel‐based wound dressings have attracted sustained interest due to their ability to support fibroblast proliferation and maintain a moist healing environment; however, achieving simultaneous antibacterial efficacy, mechanical robustness, and biocompatibility. In response to these limitations, we report a multifunctional antibacterial hydrogel incorporating blue‐emissive lanthanum‐functionalized nitrogen‐doped carbon dots (La, N‐CDs), synthesized via a green and cost‐effective hydrothermal route using citric acid, urea, and lanthanum nitrate. Lanthanum coordination with surface oxygen‐ and nitrogen‐containing groups enhances the antibacterial activity of N‐CDs by promoting stronger interactions with bacterial cell envelope, by strengthening coordination‐associated antibacterial mechanism that surpasses undoped counterparts. The La, N‐CDs were integrated into a poly(vinyl alcohol)–borax hydrogel network, further reinforced with dipotassium glycyrrhizinate as a multifunctional cross‐linker. Abundant hydrophilic functional groups from both dipotassium glycyrrhizinate (DPG) and La, N‐CDs impart high surface wettability (contact angle <20°) and rapid swelling (>180%), facilitating efficient wound exudate absorption and sustained moisture retention. The resulting hydrogel exhibits acceptable cytocompatibility (cell viability >80%) alongside pronounced antibacterial activity against *Escherichia coli* (*E. Coli*) and *Staphylococcus aureus* (*S. aureus*). Collectively, this work establishes for rare‐earth‐functionalized carbon dot hydrogels for antibacterial performance, structural integrity, and biological compatibility.

## Graphical abstract

The schematic depicts a dynamically cross‐linked PVA hydrogel network in which reversible borate ester bonds and hydrogen‐bonding interactions integrate lanthanum‐doped carbon dots (La‐CDs) and dipotassium glycyrrhizinate (DPG) to form a mechanically robust, multifunctional matrix. The hierarchical porous architecture enables sustained antibacterial activity and a moisture‐retentive wound microenvironment, resulting in accelerated tissue regeneration in an in vivo wound‐healing model.

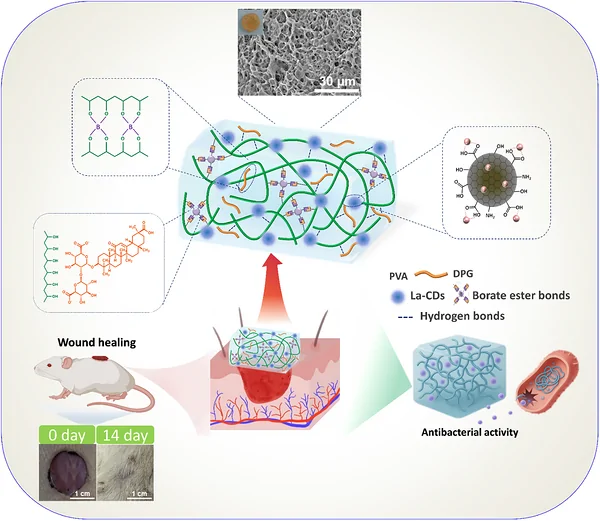

## 1 Introduction

Skin functions as a complex biological barrier that regulates hydration, provides mechanical protection, and restricts microbial invasion. Disruption of this barrier exposes underlying tissue to pathogenic microorganisms, frequently resulting in infection‐driven inflammation that impedes wound closure and compromises tissue regeneration [1, 2]. Although antibiotics remain the clinical standard for infection control, their extensive use has accelerated antimicrobial resistance, reduced therapeutic efficacy, and prolonged healing timelines [3, 4]. These challenges highlight a fundamental limitation of antibiotic‐dependent wound care and motivate the development of alternative wound dressings that provide intrinsic antibacterial protection while remaining biologically compatible. Rare earth elements (REEs) have emerged as unconventional yet increasingly investigated candidates for biomedical applications due to their unique coordination chemistry and biological activity [5, 6, 7]. Several REE‐based compounds exhibit anticoagulant, anti‐inflammatory, burn‐healing, and antibacterial effects [8, 9, 10, 11]. Among them, lanthanum (La) is a light REE of particular interest owing to its relative abundance, low cost, and reported ability to influence cellular processes. Lanthanum ions interact strongly with bacterial membranes and cell wall components, disrupting membrane integrity and interfering with calcium‐dependent biological functions [12, 13, 14, 15]. However, despite promising antibacterial activity, the direct use of lanthanum salts or nanoparticles is fundamentally constrained by poor stability, uncontrolled ion release, and dose‐dependent cytotoxicity, which limit their applicability in wound environments where prolonged tissue contact is unavoidable.

Carbon dots (CDs) offer a complementary materials platform characterized by ultrasmall size, high hydrophilicity, tunable surface chemistry, and favorable biocompatibility [16, 17, 18]. Their application spans bioimaging, drug delivery, biosensing, and antibacterial systems. Nonetheless, pristine CDs often exhibit insufficient antibacterial potency and variable performance under physiological conditions, largely due to their dependence on surface states and environmental interactions [19, 20, 21]. Functional modification via heteroatom doping, surface ligand engineering, or coordination with metal ions has therefore become essential to enhance CD stability and biological function. Such tunability has positioned CDs as adaptable nanostructures for biomedical design [22, 23]. Notably, coordination bonding between CDs and REEs offers a mechanistically appealing strategy to stabilize metal ions, modulate their bioavailability, and reduce cytotoxic risk while preserving antibacterial activity an approach that remains underexplored in wound dressing systems. Parallel advances in wound care have emphasized hydrogel dressings due to their ability to maintain a hydrated microenvironment, absorb exudate, and conform to irregular wound geometries [24, 25, 26]. Hydrogel performance is governed by network architecture, crosslinking density, and molecular interactions within the polymer matrix. Polyvinyl alcohol (PVA), owing to its hydrophilicity, chemical stability, and biocompatibility, is widely used in hydrogel fabrication. When crosslinked with borax, PVA forms reversible borate ester bonds that generate dynamic networks suitable for soft tissue contact [27, 28, 29]. However, conventional PVA‐based hydrogels suffer from limited mechanical robustness, lack intrinsic antibacterial functionality, and often fail to accommodate repeated skin deformation, restricting their translational relevance [30, 31, 32].

In this work, these limitations are addressed through the rational integration of coordination chemistry, supramolecular reinforcement, and matrix‐regulated antibacterial performance. A dual‐crosslinked PVA/borax network was reinforced with dipotassium glycyrrhizinate (DPG), introducing hydrogen‐bond–mediated mechanical strengthening and anti‐inflammatory functionality. Concurrently, lanthanum‐coordinated nitrogen‐doped carbon dots (La, N‐CDs) were synthesized via a hydrothermal route using environmentally benign precursors, yielding a coordination‐stabilized antibacterial agent with reduced cytotoxic liability. Incorporation of La, N‐CDs into the PVA/borax/DPG (PBD) hydrogel via freeze–thaw processing enabled modulation of network density, swelling behavior, and antibacterial performance. This study demonstrates that embedding coordination‐stabilized La, N‐CDs within a dynamically crosslinked hydrogel matrix enables effective antibacterial activity while maintaining cytocompatibility, hemocompatibility, and tissue compatibility. By decoupling antibacterial efficacy from mammalian toxicity and simultaneously achieving skin‐compliant mechanics, the La, N‐CDs/PBD hydrogel establishes a mechanistically grounded and translationally relevant strategy for advanced wound dressing design.

The novelty of this design does not arise from the individual use of CDs, La^3^^+^ ions, or PVA‐based hydrogels, which have been separately reported. Instead, the key distinction is the coordination‐associated integration of La^3^^+^ with N‐CD surface functionalities, followed by immobilization within a DPG‐reinforced dynamic PVA–borax network. This design differs from conventional CD‐loaded hydrogels, where CDs often act as passive fillers, and from metal‐ion‐loaded hydrogels, where antibacterial activity commonly depends on direct free‐ion release [33]. Here, La, N‐CDs serve as a coordination‐modified antibacterial interface, while the PVA–borax–DPG matrix influences swelling, release behavior, and mechanical compliance.

## 2 Results and Discussion

### 2.1 Structural and Functional Group Analysis

Lanthanum ions (La^3^^+^) exhibit strong Lewis acidity and a pronounced affinity toward oxygen‐ and nitrogen‐containing ligands [34], enabling robust coordination with surface functionalities on nitrogen‐doped carbon dots (N‐CDs). This coordination propensity provides the chemical basis for deliberate surface modification rather than bulk structural alteration. As illustrated in Figure 1a, La^3^^+^ ions coordinate with surface carboxyl, hydroxyl, and amine groups on N‐CDs, yielding La, N‐CDs through multidentate metal–ligand interactions rather than simple electrostatic adsorption. Such surface‐confined coordination is expected to preserve the carbon core while selectively modulating interfacial chemistry. Consistent with this expectation, high‐resolution transmission electron microscopy (HR‐TEM) images of pristine N‐CDs (Figure S1) and La, N‐CDs (Figure 1b) reveal well‐defined spherical nanoparticles with an average diameter of ∼5 nm. Both samples exhibit clear lattice fringes with an interplanar spacing of ∼0.21 nm, characteristic of graphitic carbon domains, confirming that La^3^^+^ coordination does not disrupt the intrinsic carbon framework. Importantly, no observable aggregation is detected, indicating that La incorporation preserves colloidal stability, a prerequisite for controlled dispersion and sustained release from hydrogel matrices. To further elucidate the nature of surface interactions implied by the schematic and microscopic observations, FT‐IR spectroscopy was employed to probe functional group involvement.

**FIGURE 1:**
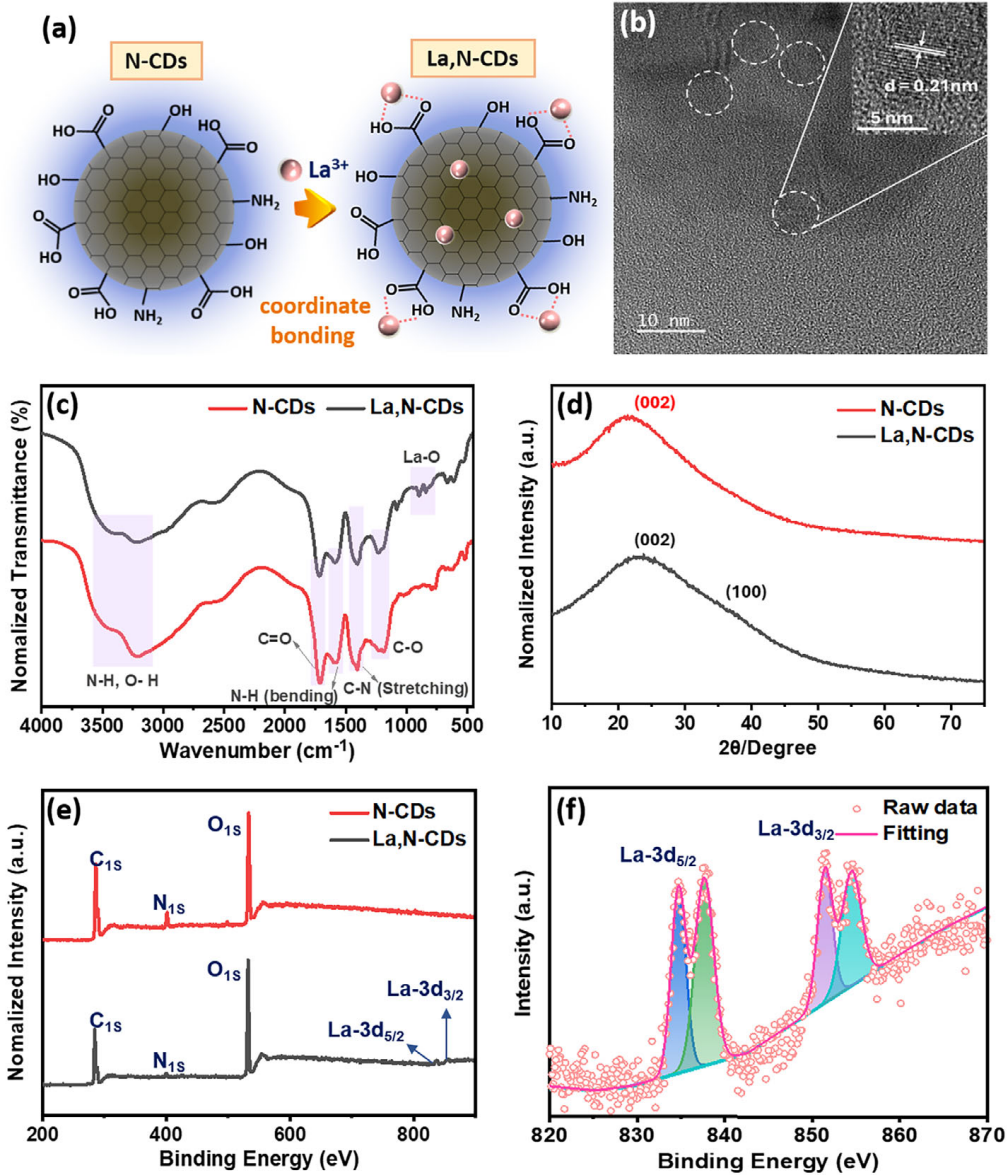
Structural and chemical characterization of La, N‐CDs. (a) Schematic illustration of N‐CDs and La, N‐CDs, highlighting coordination bonding between La^3^^+^ ions and surface functional groups. (b) HR‐TEM image of La, N‐CDs showing uniform spherical morphology and clear lattice fringes. (c) FT‐IR spectra of N‐CDs and La, N‐CDs, indicating surface functional groups and coordination‐induced changes. (d) XRD patterns of N‐CDs and La, N‐CDs, confirming the graphitic carbon structure. (e) XPS survey spectra of N‐CDs and La, N‐CDs, showing elemental composition. (f) High‐resolution La 3d XPS spectrum of La,N‐CDs, confirming the trivalent state of lanthanum.

The FT‐IR spectra (Figure 1c) display a broad band at 3100–3500 cm^−^^1^ corresponding to overlapping O–H and N–H stretching vibrations, while the absorption at ∼1710 cm^−^^1^ arises from C = O stretching of carboxyl groups. The band at ∼1570 cm^−^^1^ is assigned to amide (–NH) bending, with additional features at ∼1400 cm^−^^1^ (C–N stretching) and ∼1180 cm^−^^1^ (C–O stretching) [35, 36]. Importantly, upon lanthanum coordination, La,N‐CDs exhibit new low‐frequency vibrational features in the 750–895 cm^−^^1^ region [37], attributable to La–O coordination modes rather than La–H bonds. The emergence of these modes provides direct spectroscopic evidence of metal–ligand complexation between La^3^^+^ and oxygenated surface groups, reinforcing the coordination‐driven modification pathway proposed in Figure 1a. Structural consequences of this surface coordination were next examined using x‐ray diffraction. XRD patterns (Figure 1d) of both N‐CDs and La,N‐CDs show a broad diffraction peak centered at 2θ ≈ 23.4°, corresponding to the (002) plane of turbostratic graphitic carbon [18, 20, 38]. This diffuse feature reflects short‐range ordering and disordered layer stacking, which is typical of carbon dots derived from heteroatom‐rich precursors. Notably, upon La coordination, an additional weak diffraction feature appears at ∼40°, indexed to the (100) plane, suggesting subtle rearrangement of in‐plane carbon domains. The appearance of this feature without the emergence of sharp crystalline peaks indicates that coordination induces local structural reorganization rather than crystallization or phase segregation, in agreement with the preserved morphology observed by HR‐TEM.

To directly correlate these spectroscopic and structural observations with surface chemical states, x‐ray photoelectron spectroscopy (XPS) was conducted. XPS survey spectra (Figure 1e) of N‐CDs show dominant C1s (285 eV), N1s (400 eV), and O1s (531 eV) signals, confirming their heteroatom‐doped carbon composition [16, 17]. High‐resolution C1s spectra (Figure S2a) deconvolute into C–C/C = C (284.2 eV), C–O/C–N (285.4 eV), and O = C–O (288.0 eV) components, while N1s spectra (Figure S2b) reveal pyrrolic/amine‐type N–H (399.5 eV) and graphitic or C–N species (401.1 eV). The O1s spectrum (Figure S2c) contains contributions from hydroxyl (531.4 eV) and carbonyl/ether oxygen (532.8 eV). La,N‐CDs retain these features (Figure S2d–f), indicating preservation of surface chemistry upon metal coordination [38, 39]. Crucially, beyond these shared characteristics, the high‐resolution La 3d spectrum (Figure 1f) displays characteristic La^3^^+^ spin–orbit doublets at 834.7/837.6 eV (La 3d_5_/_2_) and 851.5/854.5 eV (La 3d_3_/_2_), unequivocally confirming the trivalent oxidation state [40] and successful incorporation of lanthanum into the carbon dot architecture. Finally, the spatial distribution of elements was examined to validate the chemical conclusions drawn from XPS. Energy‐dispersive x‐ray spectroscopy (EDS) mapping (Figure S3) further corroborates these findings, showing homogeneous distributions of C, N, and O in N‐CDs (Figure S3a), and the additional uniform presence of La in La, N‐CDs (Figure S3b). The consistency between XPS, FTIR, and EDS analyses supports coordination‐associated incorporation of La^3^^+^ within the N‐CD surface environment, rather than simple macroscopic phase separation.

Building on the coordination‐driven surface modification and preserved carbon framework discussed above, the optical properties of N‐CDs and La,N‐CDs were examined to assess the influence of La^3^^+^ coordination on electronic structure without disrupting intrinsic emissive behavior. UV–visible absorption spectra (Figure S4a,c) reveal distinct bands at ∼210 nm and ∼320 nm for both materials. Consistent with the retained sp^2^‐conjugated carbon domains observed by HR‐TEM and XRD (Figure 1b,d), the 210 nm band is assigned to π–π* transitions of C = C bonds, while the 320 nm feature arises from n–π* transitions of C = O groups and surface‐related electronic transitions associated with oxygen‐ and nitrogen‐containing functionalities, corroborating the heteroatom‐rich surface chemistry confirmed by FT‐IR and XPS analyses. Fluorescence spectra show strong emission maxima at 456 nm for N‐CDs and 453 nm for La,N‐CDs under optimal excitation. As shown in Figure S4b,d, increasing the excitation wavelength from 340 to 430 nm produces a systematic red shift in emission accompanied by an initial increase and subsequent decrease in fluorescence intensity. This excitation‐dependent behavior reflects heterogeneous emissive states associated with surface defects and functional group–derived energy levels generated during hydrothermal carbonization. Importantly, the persistence of excitation‐dependent fluorescence in La,N‐CDs indicates that La^3^^+^ coordination, while engaging surface oxygen and nitrogen sites, does not quench defect‐mediated emissive pathways. Instead, La^3^^+^ functions as an interfacial electronic modulator, subtly perturbing surface energy levels while preserving the intrinsic photophysical characteristics of the carbon dots, consistent with the coordination‐driven modification strategy established earlier.

### 2.2 Antibacterial Characteristics of La, N‐CDs

Following the coordination‐driven surface modification and preserved interfacial functionality established above, the antibacterial performance of N‐CDs and La, N‐CDs was evaluated using inhibition zone measurements. The antibacterial action of La, N‐CDs against *Escherichia coli* (*E. coli*) and *Staphylococcus aureus* (*S. aureus*) is schematically illustrated in Figure 2a. Pristine N‐CDs produced negligible inhibition zones against both strains (Figure 2b), indicating minimal intrinsic antibacterial activity in the absence of coordinated La species. In contrast, lanthanum nitrate (La(NO_3_)_3_) generated inhibition zones of approximately 1.5 cm for both *E. coli* and *S. aureus*, consistent with the antibacterial activity of free La^3^^+^ ions. Notably, La, N‐CDs exhibited larger inhibition zones than the tested La(NO_3_)_3_ condition, with diameters of 2.7 cm against *E. coli* and 1.9 cm against *S. aureus* (Figure 2b). This result suggests that the N‐CD surface environment contributes to the antibacterial presentation of La species. Rather than attributing the effect solely to free La^3^^+^ ions, the enhanced inhibition may arise from the combined contribution of La‐associated coordination sites and the oxygen‐/nitrogen‐rich surface functionalities, which can facilitate contact with bacterial cell envelopes and promote membrane disruption. The weaker activity against S. aureus compared with *E. coli* may be related to differences in bacterial envelope structure, particularly the thicker peptidoglycan layer of Gram‐positive bacteria, which can reduce nanoparticle–membrane interaction. These results indicate that the antibacterial performance of La, N‐CDs is influenced by both surface coordination chemistry and bacterial cell‐wall architecture.

**FIGURE 2:**
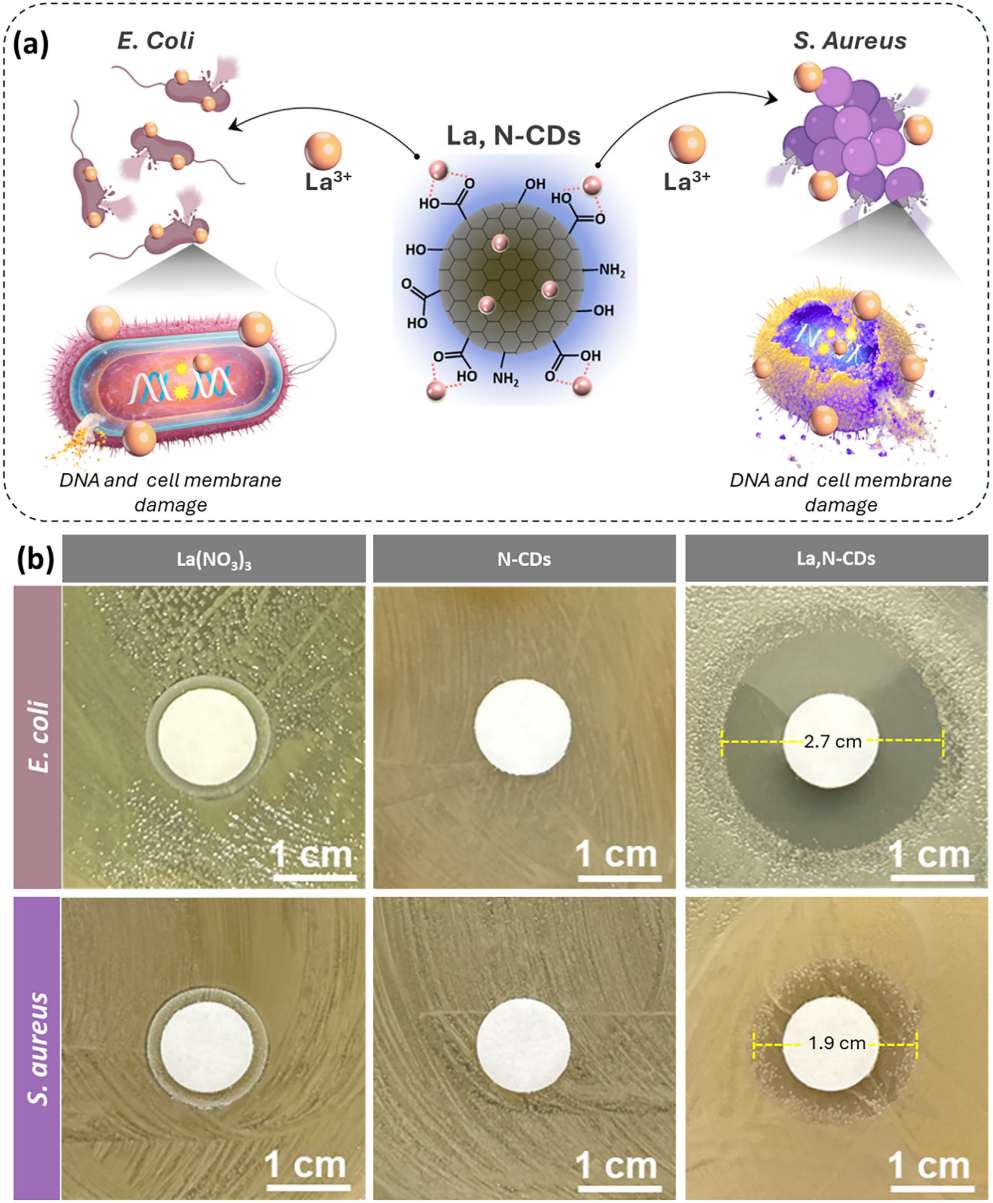
Antibacterial mechanism and inhibition zone analysis of La,N‐CDs. (a) Schematic illustration of the proposed antibacterial action of La,N‐CDs against *E. coli* and *S. aureus*, highlighting the proposed surface coordination–associated interaction with bacterial envelopes and membrane disruption, and membrane damage and loss of bacterial structural integrity. (b) Representative inhibition zone images of *E. coli* and *S. aureus* treated with La(NO_3_)_3_, N‐CDs, and La,N‐CDs. Pristine N‐CDs show negligible antibacterial activity, while La(NO_3_)_3_ produces moderate inhibition. La,N‐CDs exhibit significantly enhanced antibacterial efficacy, with inhibition zone diameters of ∼2.7 cm for *E. coli* and ∼1.9 cm for *S. aureus*.

### 2.3 Concentration‐Dependent Cellular Response to La,N‐CDs

The cytocompatibility of La,N‐CDs was systematically evaluated by combining morphological observation and quantitative cell‐viability analysis across a wide concentration range (Figure S5a). Live‐cell microscopy reveals that cells in the negative control maintain normal spindle‐like morphology before and after incubation, whereas high concentrations of La,N‐CDs (10%–2%) induce pronounced morphological disruption, cell rounding, and reduced cell density, indicative of severe cytotoxic stress. This concentration‐dependent deterioration is consistent with excessive exposure to La‐associated ionic interactions and surface‐reactive functional groups, which can compromise membrane integrity and intracellular homeostasis. Quantitative viability data further corroborates these observations. At 10%–2% concentrations, cell viability sharply decreases to ∼14%–16%, confirming that high La,N‐CDs loading exceeds the cellular tolerance threshold. A partial recovery is observed at 1%, yet viability remains below 25%, indicating persistent cytotoxic effects. In contrast, the 0.1% La,N‐CDs group exhibits cell viability approaching that of the negative control (∼97%), with preserved morphology comparable to untreated cells (Figure S5b). This sharply contrasting response highlights a narrow but well‐defined biocompatible concentration window for La,N‐CDs. Importantly, positive control shows significantly reduced viability, validating the sensitivity of the assay. These results demonstrate that while La,N‐CDs possess strong bioactivity, their biological response is highly dose‐dependent. At appropriately low concentrations, La, N‐CDs maintain excellent cytocompatibility, supporting their safe integration into hydrogel matrices for wound‐dressing applications. Conversely, direct high‐dose exposure is cytotoxic, underscoring the necessity of controlled loading and sustained‐release strategies such as hydrogel immobilization to harness antibacterial efficacy without compromising cellular viability.

### 2.4 Structure–Property Engineering of Antibacterial Hydrogels

#### 2.4.1 Mechanical Optimization via DPG‐mediated Network Regulation

Building on the coordination‐engineered antibacterial functionality of La,N‐CDs, mechanically compliant hydrogels were developed to serve as conformable wound patches capable of maintaining intimate contact with dynamic skin surfaces. To optimize network mechanics, PB hydrogels were incorporated with varying concentrations of dipotassium glycyrrhizinate (DPG; 0.1–0.5 wt%), followed by tensile testing. As shown in Figure S6a, the pristine PB hydrogel exhibited a limited maximum stress of 22 kPa and a strain of 110%, reflecting a relatively loose borate‐crosslinked network. Progressive incorporation of DPG significantly enhanced mechanical performance, with the hydrogel containing 0.3 wt% DPG achieving an optimal balance of strength (100 kPa) and extensibility (169%). This improvement is attributed to reinforced hydrogen bonding interactions between PVA chains and DPG, which promote effective stress transfer without excessive network rigidification. At higher DPG content (0.5 wt%), the maximum stress decreased to 60 kPa and the strain dropped to 161%, indicating mechanical deterioration due to over‐crosslinking. Excessive hydrogen bonding introduces network heterogeneity and local stress concentration, ultimately compromising mechanical integrity. These results underscore the importance of finely tuning supramolecular interactions to achieve mechanically robust yet flexible wound dressings.

#### 2.4.2 Molecular Interactions and Crystallinity Modulation

The molecular basis of network evolution was examined by FT‐IR spectroscopy (Figure S6b,c). The PB hydrogel displays a characteristic borate peak at 1262 cm^−^^1^, corresponding to asymmetric B–O–C stretching, confirming the formation of covalent borate ester crosslinks between borax and PVA hydroxyl groups. Upon incorporation of DPG, a distinct secondary alcohol absorption peak emerges at 1050 cm^−^^1^, verifying the successful integration of DPG into the PB network. Concurrently, the broad O–H stretching band shifts from 3261 to 3284 cm^−^^1^ with increasing DPG content (Figure S6c), providing clear evidence of strengthened hydrogen bonding interactions between PVA and DPG.

XRD patterns (Figure S6d) further reveal that semi‐crystalline PVA exhibits diffraction peaks at 19.6°, 22.7°, and 42.8° [41]. DPG, by contrast, shows no discernible crystalline peaks, consistent with its amorphous nature. PB hydrogels retain PVA‐like diffraction features, while the introduction of DPG progressively attenuates peak intensities, indicating disruption of PVA microcrystallite formation through intermolecular and intramolecular hydrogen bonding. This reduction in crystallinity confirms good component compatibility and contributes to enhanced flexibility and network stability.

#### 2.4.3 Microstructural Evolution Induced by DPG, N‐CDs, and La, N‐CDs

The morphological evolution of the hydrogel network provides direct insight into the structure–function relationships governing mechanical robustness and antibacterial release behavior. As shown in Figure 3a,b, the PB hydrogel exhibits a relatively compact and less interconnected microstructure, indicative of limited crosslinking density. Upon incorporation of dipotassium glycyrrhizinate (PBD; Figure 3c), the internal network becomes markedly more open and interconnected, reflecting enhanced hydrogen‐bond‐mediated interactions that promote network expansion and flexibility. This structural transition is consistent with the observed improvement in mechanical compliance discussed earlier. Introduction of N‐CDs into the PBD matrix further modifies the pore architecture (Figure 3d), yielding a more homogeneous and highly porous structure. The uniform distribution of pores suggests effective integration of N‐CDs within the polymer network without inducing macroscopic phase separation, while simultaneously increasing the internal surface area available for interfacial interactions. Such a configuration is favorable for mass transport and supports sustained release of functional species. Notably, incorporation of La,N‐CDs results in a denser yet well‐connected porous network (Figure 3e), indicating that coordination interactions between La^3^^+^ ions and functional groups on both carbon dots and polymer chains contribute to additional physical crosslinking. This dual role of La,N‐CDs—as antibacterial agents and network modulators—distinguishes this system from conventional filler‐loaded hydrogels, where mechanical reinforcement often compromises diffusivity.

**FIGURE 3:**
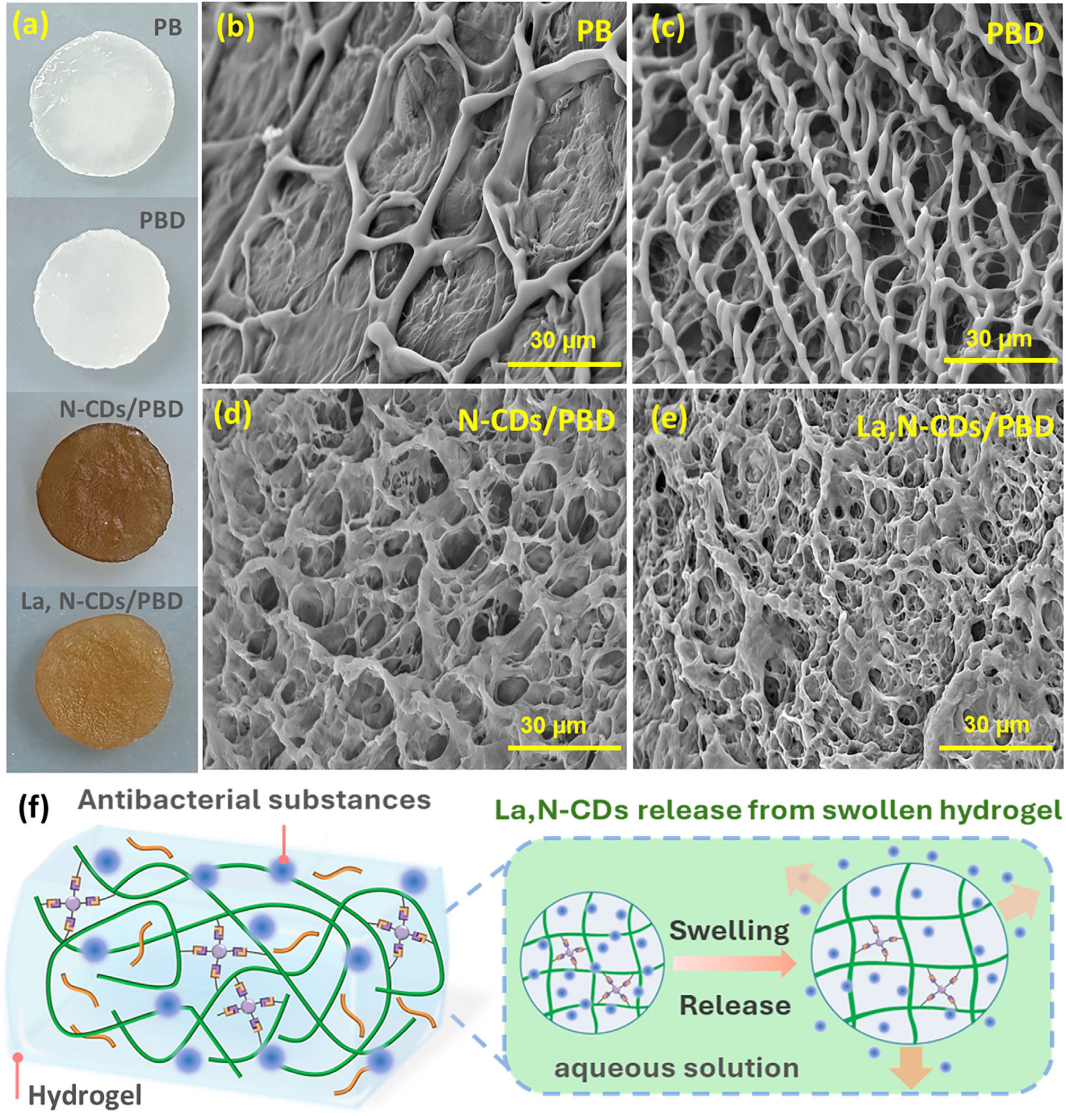
Morphology and release mechanism of La, N‐CDs–integrated hydrogels. (a) Photographs of PB, PBD, N‐CDs/PBD, and La, N‐CDs/PBD hydrogels. (b–e) SEM images showing the internal porous microstructures of PB (b), PBD (c), N‐CDs/PBD (d), and La, N‐CDs/PBD (e) hydrogels, revealing progressive network densification upon DPG incorporation and carbon dot loading. (f) Schematic illustration of the hydrogel network incorporating antibacterial agents and the swelling‐induced release of La, N‐CDs from the hydrogel matrix in aqueous environments.

Figure 3f schematically illustrates the swelling‐associated interaction between the aqueous environment and the La,N‐CDs/PBD hydrogel network. Upon hydration, water uptake expands the hydrogel matrix and may facilitate exposure of embedded La,N‐CDs at the wound‐contact interface. Therefore, the antibacterial function is interpreted as matrix‐associated and swelling‐influenced, rather than as quantitatively defined release kinetics. The high‐magnification SEM images (Figure S7) further support this structural evolution, showing a transition from compact, irregular pores in PB to a more interconnected porous architecture in La,N‐CDs/PBD. These observations suggest that La,N‐CD incorporation contributes to network reorganization and pore‐structure modulation. Taken together, the microstructural features of the La,N‐CDs/PBD hydrogel are important for its mechanical integrity, aqueous swelling response, and antibacterial performance, supporting its potential as an advanced wound dressing material.

#### 2.4.4 Skin‐Relevant Mechanical Compliance

Given that human skin undergoes continuous deformation during motion, wound dressings must tolerate large strains without mechanical failure. Tensile testing (Figure 4a) shows that incorporation of N‐CDs and La,N‐CDs reduces hydrogel stress by partially occupying PVA crystalline domains and disrupting chain alignment during freeze–thaw cycles. Simultaneously, surface functional groups on N‐CDs and La,N‐CDs promote physical crosslinking, markedly improving elongation at break. The N‐CDs/PBD and La,N‐CDs/PBD hydrogels exhibit elongations of 208% and 210%, respectively, exceeding the typical strain range of human skin (20%–180%) [42, 43]. These results demonstrate excellent conformability and mechanical suitability for wound patch applications.

**FIGURE 4:**
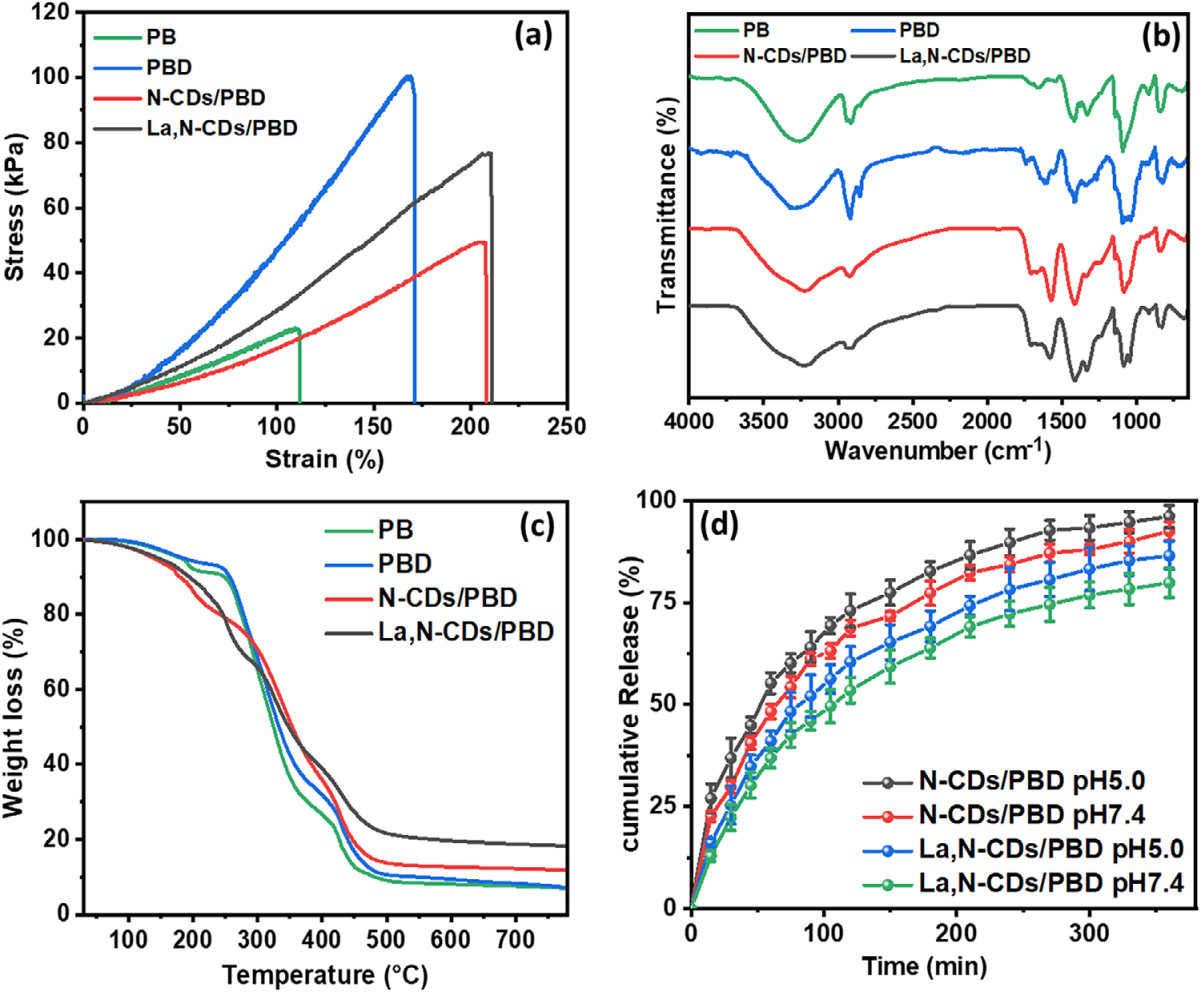
Mechanical, structural, thermal, and swelling properties of PB, PBD, N‐CDs/PBD, and La, N‐CDs/PBD hydrogels. (a) Tensile stress–strain curves showing enhanced strength and toughness after DPG and N‐CDs/La, N‐CDs incorporation. (b) FT‐IR spectra evidencing strengthened hydrogen bonding and coordination interactions within the hydrogel network. (c) TGA curves indicating improved thermal stability with functional additives. (d) Release profiles of N‐CDs and La,N‐CDs from hydrogels at pH 5.0 and 7.4, revealing pH‐responsive behavior relevant to wound environments (Data are presented as mean ± SD, *n* = 3).

#### 2.4.5 Interfacial Bonding and Thermal Stability

FT‐IR spectra of composite hydrogels (Figure 4b) reveal pronounced changes in the broad O–H band (3000–3500 cm^−^^1^) and a significant increase in the C = O peak at 1660 cm^−^^1^, confirming hydrogen bond formation between hydroxyl and carbonyl groups. The oxygen‐rich surfaces of N‐CDs and La,N‐CDs facilitate these interactions, enabling their stable integration into the PBD network Thermogravimetric analysis (Figure 4c) shows that PB hydrogels undergo three degradation stages: water evaporation (50–250°C), side‐chain hydroxyl decomposition (250–400°C), and main‐chain PVA degradation (400–700°C) [44, 45]. PBD hydrogels exhibit improved thermal stability due to strengthened hydrogen bonding. N‐CDs/PBD and La,N‐CDs/PBD hydrogels display greater early‐stage weight loss, attributed to cleavage of polymer–dot interactions, but higher residual weights (11.8% and 18.3%, respectively) compared to PB (6.9%) and PBD (7.2%), indicating enhanced thermal robustness from DPG and carbon dot incorporation.

#### 2.4.6 Wettability and pH‐Responsive Swelling Behavior

Contact angle measurements (Figure S8a) show that PB hydrogels are highly hydrophilic (15.7°), owing to abundant PVA hydroxyl groups. DPG addition further reduces the contact angle to 13.9° through hydrophilic glucuronic acid moieties. Incorporation of N‐CDs and La,N‐CDs decreases the contact angle to 11.2° and 9.7%, respectively, due to surface carboxyl, hydroxyl, and amino groups. La^3^^+^ coordination further enhances polarity via oxygen affinity, yielding the most hydrophilic surface. Water content analysis (Figure S8b) reveals that increasing crosslinking density reduces water uptake by restricting pore volume and diffusion space. Despite this trend, all hydrogels maintain water contents exceeding 80%, ensuring adequate moisture retention for wound healing.

Controlled release is critical for antibacterial wound dressings. Release profiles of N‐CDs and La,N‐CDs from hydrogels (Figure 4d) show an initial rapid release within 2 h, followed by sustained release beyond 2.5 h. Under identical conditions, N‐CDs/PBD hydrogels exhibit higher cumulative release than La,N‐CDs/PBD hydrogels due to larger pore sizes, confirming that denser networks slow diffusion. Release rates decrease with increasing pH, demonstrating pH‐responsive behavior aligned with wound microenvironment regulation. Swelling studies (Figure S9) show rapid initial water uptake followed by equilibrium. Swelling is higher at pH 5.0 than at pH 7.4, likely due to protonation‐induced electrostatic repulsion and network expansion. La,N‐CDs/PBD hydrogels exhibit lower swelling than N‐CDs/PBD hydrogels at the same pH, consistent with a denser network and smaller pore structure observed by SEM. These swelling results suggest that the hydrogel matrix may influence the exposure and diffusion of embedded La,N‐CDs under aqueous conditions.

#### 2.4.7 Small‐Angle X‐Ray Scattering

Small‐angle x‐ray scattering (SAXS) was used to elucidate the temperature‐dependent nanoscale structural evolution of the hydrogel networks, focusing on interchain spacing and network reversibility. SAXS profiles of PB, PBD, N‐CDs/PBD, and La,N‐CDs/PBD hydrogels were collected between 25°C and 55°C to correlate thermal stimuli with polymer network dynamics (Figure 5). As shown in Figure 5a–d, all hydrogels display a progressive shift of scattering curves toward lower scattering vectors (q) with increasing temperature, corresponding to an increased correlation length (*r* = 2π/q). This trend reflects thermally induced weakening of hydrogen bonding and borate ester interactions, enhanced water mobility, and partial expansion of the polymer network. Upon cooling back to 25°C, the curves shift toward higher q values, indicating partial recovery of interchain proximity through reformation of reversible crosslinks. Clear differences in reversibility are observed among samples. PB and PBD hydrogels exhibit pronounced hysteresis, suggesting limited structural recovery. Incorporation of N‐CDs improves reversibility, consistent with additional hydrogen‐bonding interactions. Notably, the La, N‐CDs/PBD hydrogel shows the smallest q‐shift and the closest overlap with its initial 25°C profile after thermal cycling (Figure 5d), evidencing superior network stability. This behavior is attributed to La^3^^+^‐mediated coordination interactions that reinforce the crosslinking framework while retaining dynamic responsiveness, as schematically illustrated in Figure 5e. Such a thermally stable yet reversible nanoscale architecture is critical for wound dressing applications, enabling the La, N‐CDs/PBD hydrogel to maintain structural integrity under physiological temperature fluctuations while supporting controlled swelling and functional release. Further compressive and rheological analyses will be useful to expand the understanding of hydrogel behavior under wound‐relevant dynamic mechanical conditions.

**FIGURE 5:**
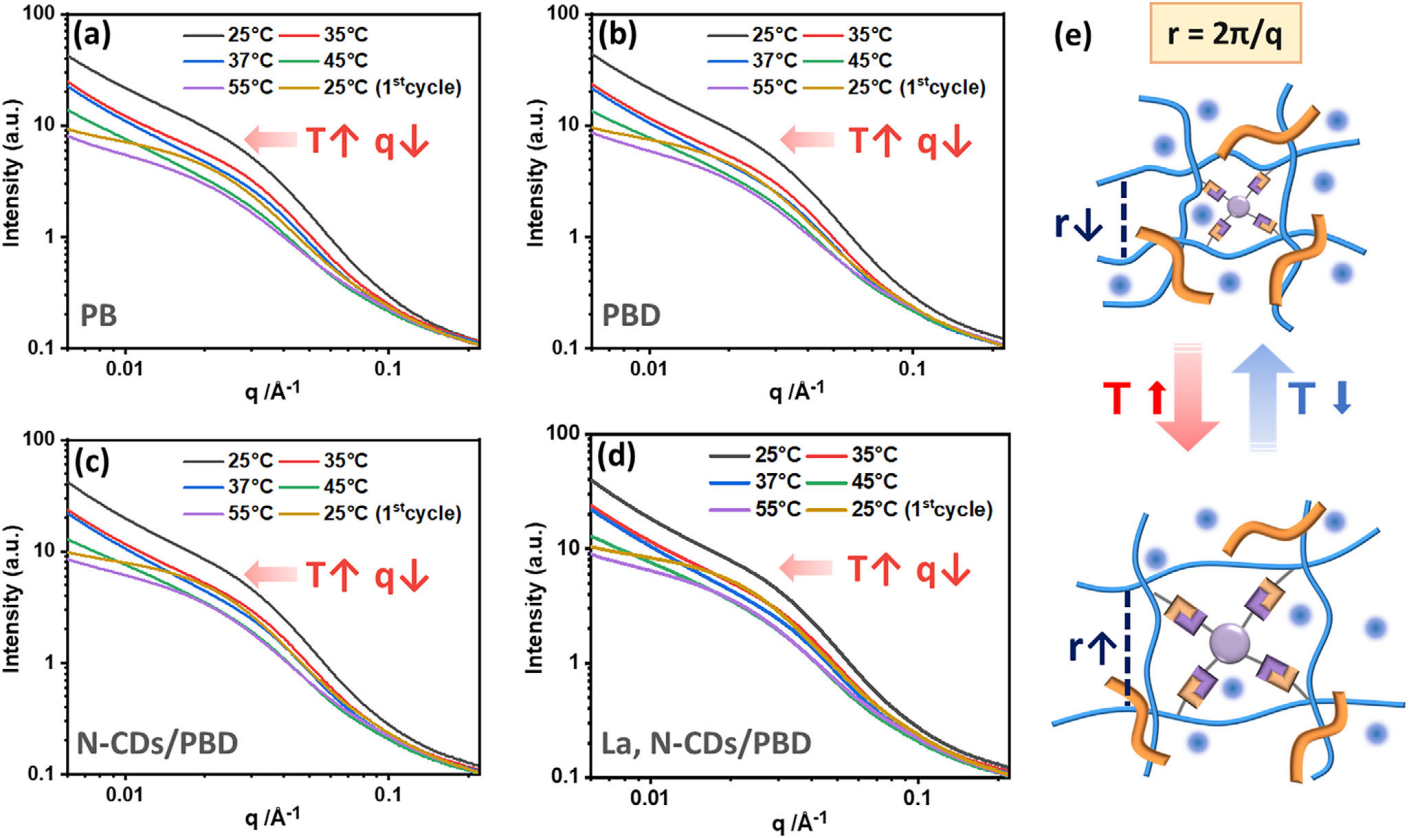
Temperature‐dependent SAXS profiles of (a) PB, (b) PBD, (c) N‐CDs/PBD, and (d) La, N‐CDs/PBD hydrogels recorded at 25°C–55°C and after cooling back to 25°C. The shift of scattering curves toward lower q with increasing temperature indicates thermally induced expansion of interchain spacing (*r* = 2π/q), while recovery upon cooling reflects reversible network reorganization. (e) Schematic illustration of temperature‐driven contraction/expansion of polymer chains and enhanced structural stability in La, N‐CDs/PBD due to strengthened crosslinking interactions.

### 2.5 Antibacterial Performance and Biocompatibility of La, N‐CD–Integrated Hydrogels

#### 2.5.1 Bacteriostatic Activity and Release‐Enabled Antibacterial Function

Consistent with the coordination‐engineered antibacterial functionality of La, N‐CDs, and their controlled release from the hydrogel network established earlier, the bacteriostatic performance of the hydrogels was evaluated using *E. coli* and *S. aureus* as representative Gram‐negative and Gram‐positive strains. As shown in Figure 6a, the PBD and N‐CDs/PBD hydrogels exhibit no discernible inhibition zones, indicating that the polymeric network and carbon dots alone do not impart effective antibacterial activity under the tested conditions. In contrast, the La,N‐CDs/PBD hydrogel forms pronounced inhibition zones against both E. coli and S. aureus, with diameters of 1.7 cm and 1.5 cm, respectively. The emergence of clear inhibition zones confirms that La, N‐CDs are effectively released from the hydrogel matrix and retain their antibacterial functionality following incorporation. This observation is consistent with the pH‐responsive and structure‐dependent release behavior discussed earlier, where La,N‐CDs/PBD hydrogels exhibited sustained release governed by crosslinking density and pore architecture. The antibacterial performance therefore arises from a combination of controlled La,N‐CD availability and preserved surface coordination chemistry, rather than from passive polymer effects. To provide additional bacterial‐count information beyond the inhibition‐zone assay, a CFU plate assay was performed after contact with the La,N‐CDs/PBD hydrogel under the tested conditions. Representative agar plate images of E. coli and S. aureus are provided in Figure S10, showing both front‐side and back‐side views of the plates. The measured CFU values for La,N‐CDs/PBD were 2.1 × 107 CFU mL–1 for *E. coli* and 2.0 × 107 CFU mL–1 for *S. aureus*. These CFU data provide supplementary bacterial‐count information and complement the agar diffusion observations in evaluating the antibacterial response of the La,N‐CDs/PBD hydrogel.

**FIGURE 6:**
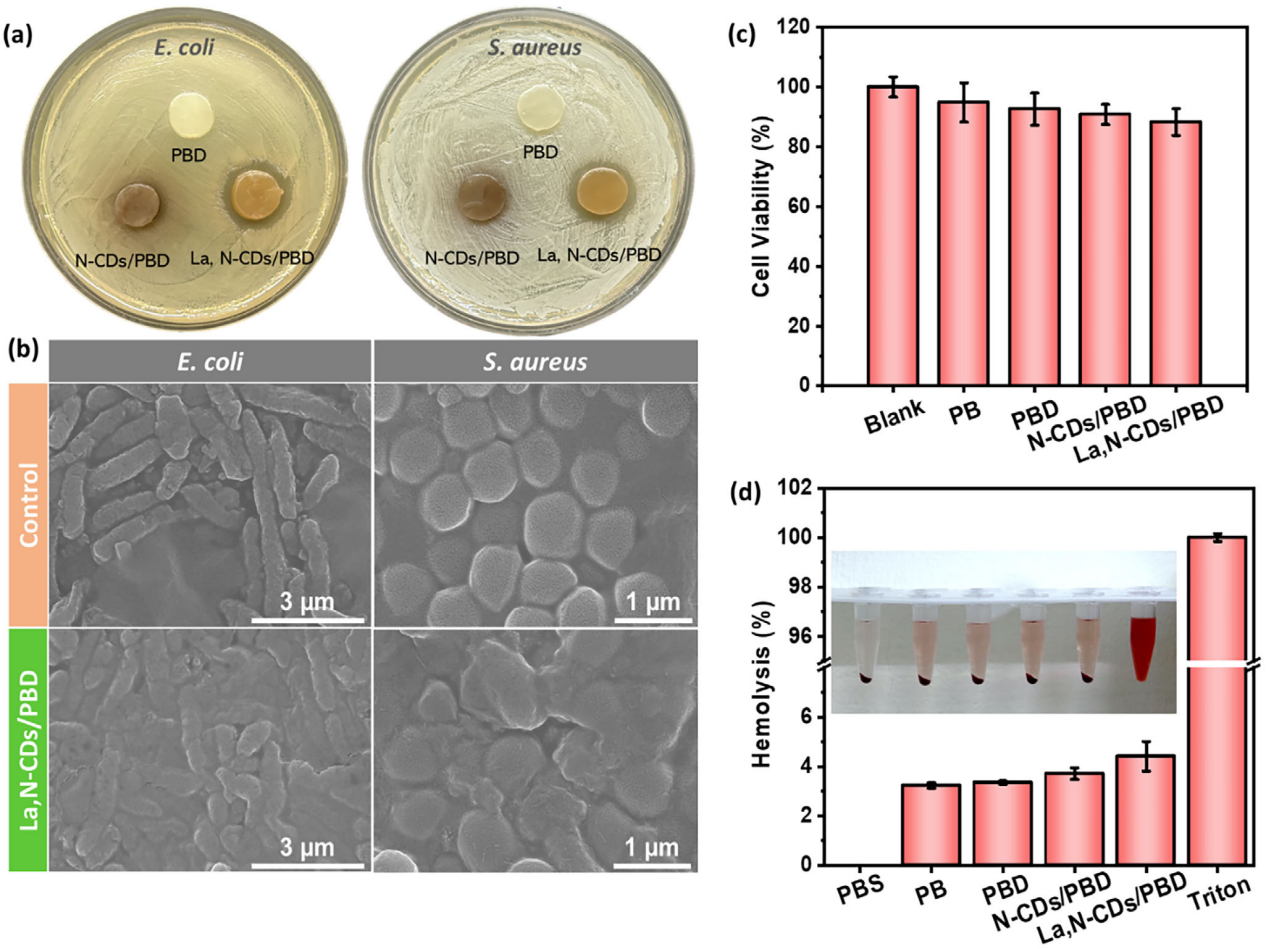
In vitro antibacterial performance and biocompatibility analysis of PB, PBD, N‐CDs/PBD and La, N‐CDs/PBD hydrogels (Data are presented as mean ± SD, *n* = 3). (a) Photograph of inhibition zones of hydrogels against *E. Coli* and *S. aureus* after incubating at 37°C for PBD, N‐CDs/PBD, and La, N‐CDs/PBD hydrogel. (b) SEM images of *E. Coli* and *S. aureus* co‐incubation with La, N‐CDs/PBD hydrogels and the control condition. (c) Cell viability of L929 cells co‐incubated with hydrogels for 24 h, and (d) Hemolysis rate analysis, the inset picture shows the solution photos of different hydrogels co‐incubated with red blood cells.

#### 2.5.2 Bacterial Morphology and Antibacterial Mechanism

To gain insight into the antibacterial action of the La, N‐CDs/PBD hydrogel, scanning electron microscopy (SEM) was used to examine bacterial morphology before and after treatment. As shown in Figure 6b, untreated *E. coli* and *S. aureus* in the control group display intact cell walls and well‐defined cellular structures. In contrast, bacteria exposed to the La,N‐CDs/PBD hydrogel exhibit pronounced morphological deformation, including surface collapse, membrane disruption, and loss of structural integrity. These observations indicate that the incorporation of La, N‐CDs induces significant damage to bacterial envelops. The observed membrane disruption is consistent with the coordination‐associated surface chemistry of La,N‐CDs discussed earlier. La‐associated surface sites and oxygen‐/nitrogen‐containing functional groups may facilitate interaction with bacterial cell envelopes, thereby weakening membrane stability and promoting structural damage [46, 47]. The antibacterial effect may also be influenced by differences in bacterial cell‐wall architecture, particularly the thicker peptidoglycan layer of Gram‐positive S. aureus, which can reduce nanoparticle–membrane interaction compared with Gram‐negative *E. coli*. Although SEM observations support a membrane‐disruptive antibacterial effect, direct bacterial live/dead staining was not performed in the present study. Therefore, the proposed antibacterial mechanism should be interpreted as morphology‐supported evidence rather than direct viability‐mapping evidence.

#### 2.5.3 Cytocompatibility and Hemocompatibility Assessment

Beyond antibacterial efficacy, biocompatibility is a critical requirement for wound dressing applications, as hydrogels are intended for direct contact with damaged tissue. To assess cytotoxicity, PB, PBD, N‐CDs/PBD, and La,N‐CDs/PBD hydrogels were co‐cultured with L929 fibroblast cells, and cell viability was evaluated using the MTT assay. As shown in Figure 6c, all hydrogel formulations exhibit cell viabilities exceeding 80% after 24 h of incubation, indicating low cytotoxicity. Notably, the N‐CDs/PBD and La,N‐CDs/PBD hydrogels show cell viabilities of approximately 90% and 88%, respectively. Although borax is known to exhibit toxicity at elevated concentrations, its use here as a crosslinking agent at controlled, low levels does not induce significant cytotoxic effects. These results indicate that incorporation of N‐CDs and La,N‐CDs does not compromise cytocompatibility, supporting the suitability of the composite hydrogels for biomedical use. Hemocompatibility was further evaluated through hemolysis assays as excessive red blood cell lysis can limit clinical applicability. A hemolysis rate below 5% is generally accepted as indicative of good blood compatibility. As shown in Figure 6d, the positive control (Triton) exhibits complete hemolysis (100%), whereas the negative control (PBS) shows no hemolysis (0%). In contrast, all hydrogel samples display hemolysis rates below 5%, with N‐CDs/PBD and La,N‐CDs/PBD hydrogels exhibiting hemolysis rates of 3.7% and 4.4%, respectively. Visual inspection of centrifuged blood samples further confirms these results: hydrogel‐treated samples retain a light red coloration similar to the PBS control, while the Triton group appears bright red. Collectively, this cytocompatibility and hemocompatibility results demonstrate that the La,N‐CD–integrated hydrogels combine effective antibacterial performance with acceptable biological safety, fulfilling key requirements for wound dressing applications.

#### 2.5.4 Agar Diffusion Cytotoxicity and Zone‐Resolved Biocompatibility

To rigorously assess local cytotoxicity under diffusion‐controlled conditions relevant to wound contact, the biocompatibility of the La,N‐CDs/PBD hydrogel was evaluated using the agar diffusion assay in accordance with ISO 10993‐5:2009. This method enables spatially resolved assessment of cellular responses to diffusible species released from the material, complementing the direct‐contact cytotoxicity data presented earlier. L929 fibroblast cells were used as the test model, with Teflon film and latex rubber serving as the negative and positive controls, respectively. As illustrated schematically in Figure 7a, three concentric evaluation regions were defined: the sample zone, the sample‐edge zone (0.5 cm from the sample boundary), and the outside zone (1.0 cm from the sample boundary) (Figure 7b). Representative optical images (Figure 7c) confirm that the negative control exhibits uniform cell density and normal fibroblast morphology across all zones, whereas the positive control induces a clear inhibition halo accompanied by extensive cell lysis, validating assay sensitivity. In contrast, the La, N‐CDs/PBD hydrogel shows cellular responses indistinguishable from the negative control across all zones. Fibroblasts maintain intact morphology, high confluence, and normal adhesion in the sample, sample‐edge, and outside zones, with no evidence of cell rounding, detachment, or membrane damage. According to ISO 10993‐5:2009 criteria, these observations correspond to a cytotoxicity grade of 0, confirming the absence of detectable cytotoxic effects.

**FIGURE 7:**
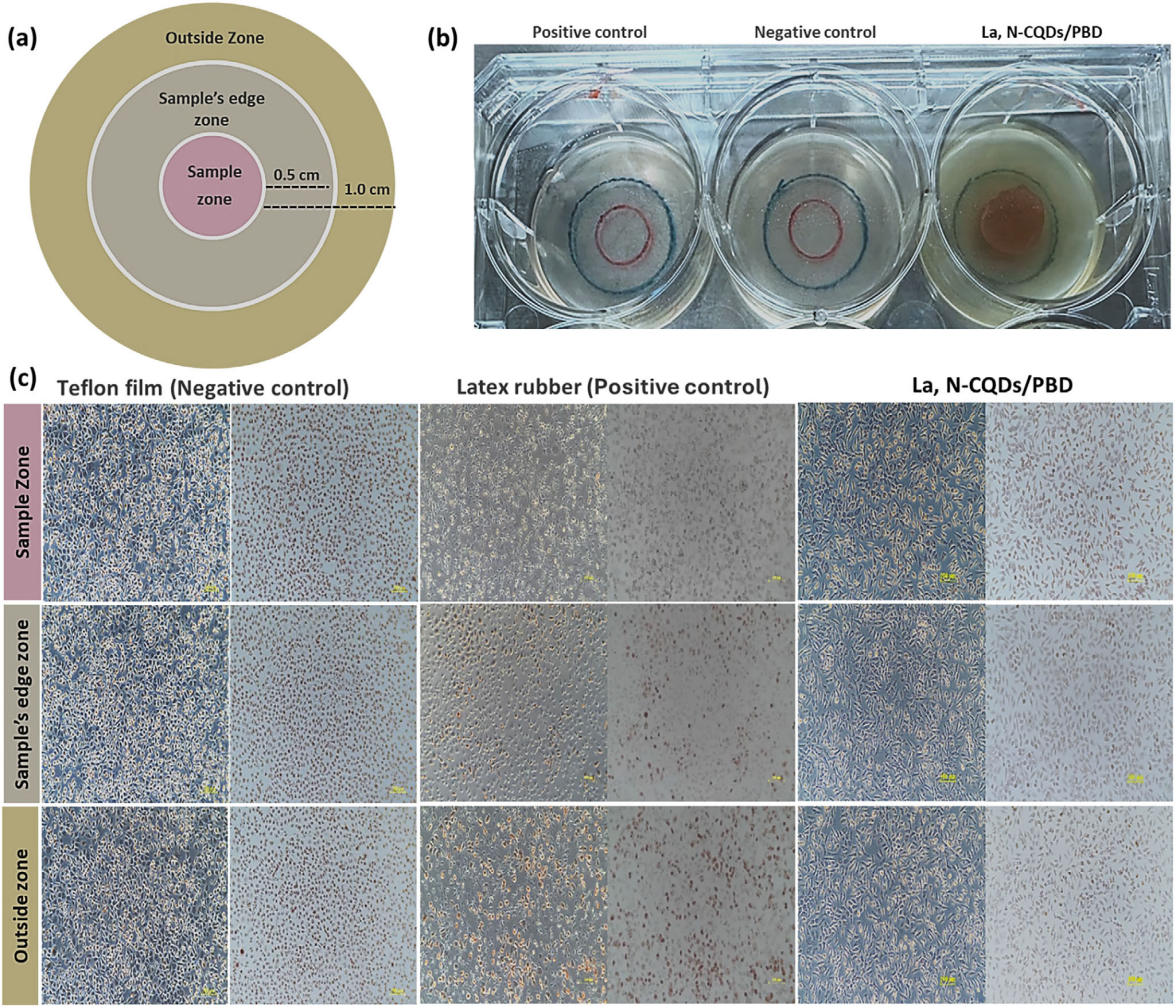
Agar diffusion cytotoxicity evaluation of La,N‐CDs/PBD hydrogel. (a) Schematic of the agar diffusion assay showing the sample, sample‐edge (0.5 cm), and outside (1.0 cm) zones. (b) Representative images of negative (Teflon film) and positive (latex rubber) controls. (c) Optical micrographs of L929 fibroblasts in the three zones after exposure to La,N‐CDs/PBD hydrogel and controls, showing no detectable cytotoxic response for La,N‐CDs/PBD (ISO 10993‐5:2009, grade 0).

This zone‐resolved biocompatibility is mechanistically significant when considered alongside the antibacterial performance discussed earlier. Although La^3^^+^ ions exhibit strong bacteriostatic activity through coordination‐mediated disruption of bacterial cell walls, their incorporation within N‐CDs and subsequent immobilization in the PBD hydrogel network effectively constrain diffusion. The absence of cytotoxic responses even in the immediate sample zone indicates that the local concentration of released La,N‐CDs remains within a biologically tolerable window—sufficient for antibacterial action yet non‐deleterious to fibroblasts. Together with the high cell viability observed in MTT assays and the low hemolysis rates reported previously, the agar diffusion results provide spatially resolved, regulatory‐aligned evidence that the La,N‐CDs/PBD hydrogel decouples antibacterial efficacy from mammalian cytotoxicity. This balance underscores the effectiveness of the coordination‐driven hydrogel design and supports its suitability for antibacterial wound dressing applications.

### 2.6 Macroscopic Wound Closure and in Vivo Wound Healing Kinetics

The therapeutic efficacy of the hydrogel dressings was evaluated using a full‐thickness excisional wound model in rats. As illustrated in Figure 8a, the control group received only artificial skin for wound fixation, without hydrogel treatment. Wound closure was monitored on days 3, 7, 10, and 14. As shown in Figure 8b, wounds in the control group gradually contracted over time, reflecting the inherent self‐healing capacity of the animal model. In contrast, all hydrogel‐treated groups exhibited accelerated wound closure at each observation point. Notably, wounds treated with the La,N‐CDs/PBD hydrogel consistently displayed smaller residual wound areas than those treated with PBD or N‐CDs/PBD hydrogels alone, indicating a superior healing‐promoting effect. Quantitative analysis of wound healing rates (Figure 8c) further substantiates this trend. By day 14, the wound‐healing rates were 91.1 ± 5.0% for the control, 92.3 ± 5.1% for PBD, 97.1 ± 4.2% for N‐CDs/PBD, and 99.1 ± 1.9% for La,N‐CDs/PBD. Statistical analysis showed that La,N‐CDs/PBD significantly enhanced wound closure compared with the control group at days 3, 7, 10, and 14, whereas the difference between N‐CDs/PBD and La,N‐CDs/PBD at day 14 was not statistically significant. The improvement from PBD to N‐CDs/PBD was larger than the additional improvement from N‐CDs/PBD to La,N‐CDs/PBD. This suggests that N‐CDs themselves play an important role in wound healing. Although N‐CDs/PBD showed weak antibacterial activity, it still promoted wound closure, likely due to its good biocompatibility, moist hydrogel environment, and oxygen‐/nitrogen‐containing surface groups, which may support cell attachment, migration, and tissue regeneration. La,N‐CDs/PBD showed the highest wound closure, but the additional improvement compared with N‐CDs/PBD was modest. This suggests that La mainly provides extra antibacterial protection rather than a large additional improvement in tissue repair. Therefore, N‐CDs/PBD may be suitable for wounds where high biocompatibility and tissue repair are most important, while La,N‐CDs/PBD may be more useful for infected or infection‐prone wounds.

**FIGURE 8:**
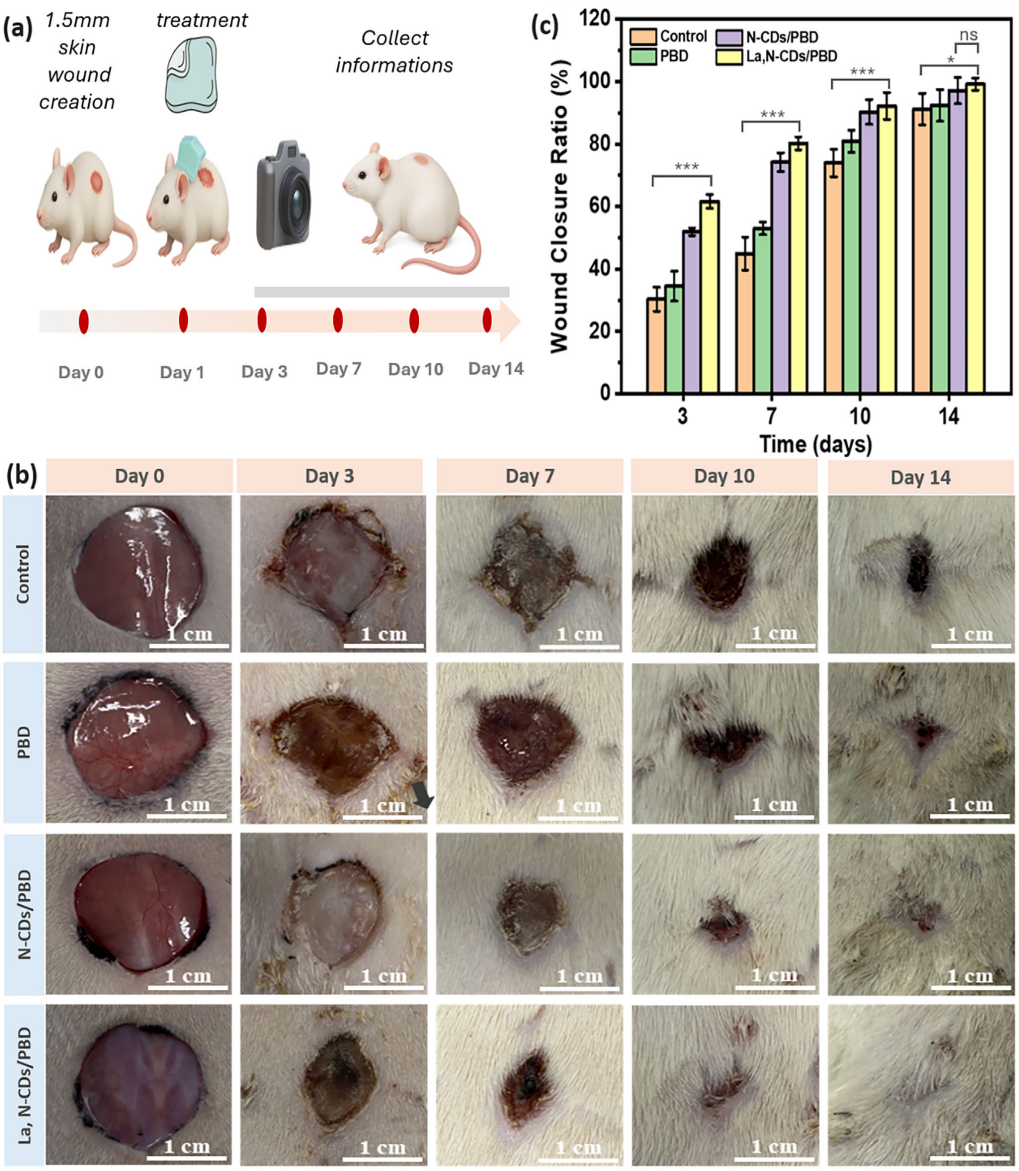
In vivo wound healing evaluation and histological analysis. (a) Schematic of the full‐thickness wound model and treatment schedule. (b) Digital images of wounds at different time intervals under various treatments. Scale bar: 1.0 cm, *n* = 3 per group. (c) Wound‐closure ratio calculated from digital images. Data are mean ± SD, *n* = 3. Statistical significance was analyzed by one‐way ANOVA followed by Tukey's test; ns, not significant; *p < 0.05; ***p < 0.001.

A comparison with representative antibacterial hydrogel wound dressings is provided in Table S1. Compared with reported systems based on antibacterial polymers, carbon dots, metal nanoparticles, nanozymes, or photothermal/antibiofilm strategies, La,N‐CDs/PBD shows a competitive combination of antibacterial response, cytocompatibility, hemocompatibility, and wound‐healing performance. In particular, the hydrogel achieved 99.1 ± 1.9% wound closure at day 14 while maintaining L929 cell viability above 80%, hemolysis below 5%, and agar diffusion cytotoxicity grade 0. Although some state‐of‐the‐art systems include more detailed antibacterial kinetic analyses, such as MIC, time‐kill, and LIVE/DEAD staining, the present La,N‐CDs/PBD hydrogel offers a non‐antibiotic rare‐earth/carbon‐dot‐based strategy with balanced antibacterial activity and biological safety.

#### 2.6.1 Histological Analysis of Tissue Regeneration

Wound healing proceeds through sequential inflammatory, granulation, and epithelialization phases [48]. To assess tissue remodeling during hydrogel‐assisted healing, regenerated skin tissues were harvested on days 7 and 14 for histological examination. Hematoxylin and eosin (H&E) staining (Figure 9) on day 7 reveals pronounced inflammatory infiltration and limited granulation tissue formation in the control group. In contrast, wounds treated with PBD hydrogel exhibit markedly reduced inflammation, consistent with the known anti‐inflammatory properties of DPG. More advanced tissue regeneration is observed in the N‐CDs/PBD and La,N‐CDs/PBD groups, characterized by early epithelial formation and emerging hair follicles. By day 14, H&E staining shows minimal epithelial regeneration in the control group, whereas hydrogel‐treated wounds display well‐developed epithelial layers and extensive hair follicle regeneration. These findings indicate that hydrogels not only mitigate inflammatory responses but also actively promote tissue reconstruction.

**FIGURE 9:**
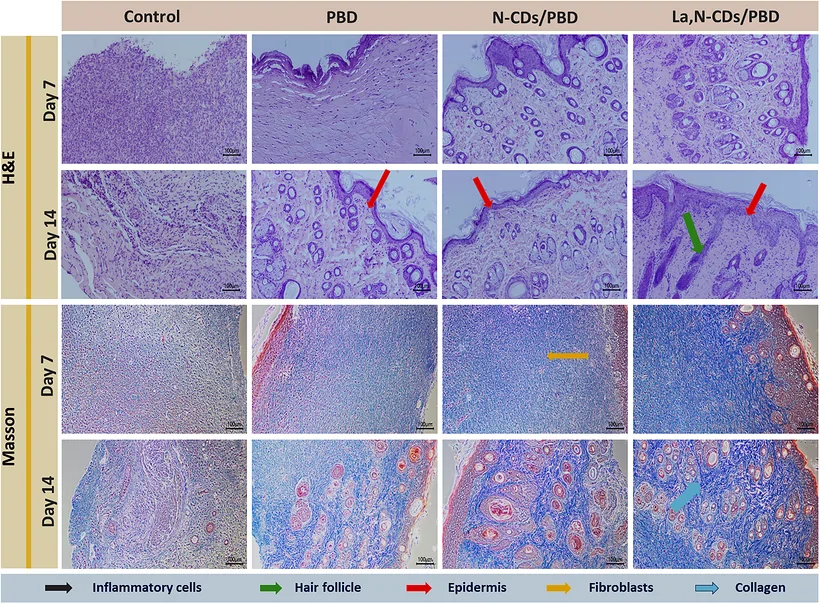
Histological analysis of wound healing. H&E and Masson's trichrome staining of wound tissues from control, PBD, N‐CDs/PBD, and La,N‐CDs/PBD groups on days 7 and 14. H&E images show inflammation, epidermal regeneration, and hair follicle formation, while Masson staining indicates collagen deposition and maturation. Scale bar: 100 µm.

#### 2.6.2 Collagen Deposition and Maturation

Collagen deposition and organization were further evaluated using Masson's trichrome staining [49, 50]. On day 7, wounds treated with the La,N‐CDs/PBD hydrogel show substantially higher collagen content compared to the control, PBD, and N‐CDs/PBD groups. By day 14, collagen deposition increases across all groups; however, the control group still exhibits sparse and disorganized collagen fibers. In contrast, the La,N‐CDs/PBD group displays dense, well‐aligned collagen networks, indicative of advanced extracellular matrix maturation (Figure S11). The enhanced collagen deposition and organization are attributed to the synergistic integration of antibacterial La,N‐CDs within the PBD hydrogel matrix, which limits bacterial burden, suppresses prolonged inflammation, and creates a microenvironment favorable for fibroblast activity and matrix remodeling. Collectively, these results confirm that the La,N‐CDs/PBD hydrogel accelerates wound closure while simultaneously improving the quality of regenerated tissue, highlighting its potential as an advanced antibacterial wound dressing.

#### 2.6.3 Limitations and Material Fate Consideration

Although the present study demonstrates short‐term wound‐healing efficacy and local biological tolerance of the La,N‐CDs/PBD hydrogel, the long‐term degradation behavior and biological fate of the hydrogel matrix and encapsulated La,N‐CDs were not directly investigated. The PVA–borax–DPG network contains reversible borate ester bonds and hydrogen‐bonding interactions, and the swelling/release results indicate that the hydrogel can interact with the aqueous wound environment. However, these results should be interpreted as preliminary material–environment interaction rather than a complete degradation or clearance profile. Future studies will include in vitro degradation in simulated wound fluid, quantitative La,N‐CD release/clearance analysis, and subcutaneous implantation to evaluate long‐term biocompatibility and residual material fate.

## 3 Conclusion

This work demonstrates the rational design of a multifunctional La,N‐CDs/PBD hydrogel dressing that integrates mechanical compliance, antibacterial activity, and biocompatibility through coordination‐ and hydrogen‐bond–mediated network engineering. The hydrogel forms a stable three‐dimensional architecture via borate ester crosslinking and dynamic hydrogen bonding between PVA and dipotassium glycyrrhizinate (DPG), enabling simultaneous enhancement of strength and extensibility. Mechanical testing and FTIR analysis confirm that optimized PVA–DPG interactions increase tensile stress from 22 to 100 kPa and strain from 110% to 169%, yielding skin‐compliant mechanics suitable for dynamic wound environments. Incorporation of La,N‐CDs introduces antibacterial functionality without compromising cytocompatibility. The coordination‐stabilized La^3^^+^ species effectively inhibit bacterial proliferation while remaining biologically tolerable, as evidenced by ISO‐compliant cytotoxicity, hemolysis, and agar diffusion assays. Importantly, the hydrogel matrix influences swelling behavior and may moderate La,N‐CDs exposure at the wound‐contact interface, balancing antibacterial efficacy with acceptable mammalian cell compatibility. In vivo wound healing studies further demonstrate accelerated wound closure, reduced inflammation, enhanced epithelial regeneration, and organized collagen deposition, highlighting the synergistic roles of DPG‐mediated anti‐inflammatory effects and La,N‐CD–driven antibacterial protection. These results establish La, N‐CDs as a promising non‐antibiotic antibacterial component for hydrogel wound dressings. These findings position La,N‐CDs/PBD hydrogels as a promising non‐antibiotic antibacterial wound‐dressing platform, although further studies on long‐term release, inflammatory regulation, and infected wound models are required to strengthen translational relevance.

## Author Contributions

S. H. Tsai and R. K. Devi contributed equally to this work by writing the original manuscript, conducting experiments, performing visualization, data curation, and English revision. M.G, G.P.C. Chien contributed to this work by validating and reviewing, and editing. C. C. Lin contributed to conceptualization and supervision. All the authors have read and agreed with the published version of the manuscript.

## Conflicts of Interest

The authors declare no conflicts of interest.

## Supporting information




**Supporting File**: adhm71608‐sup‐0001‐SuppMat.docx.

## Data Availability

The data that support the findings of this study are available from the corresponding author upon reasonable request.
