# Supplementary material for: Rare‐Earth–Functionalized Carbon Dot Hydrogels as Mechanically Robust Antibacterial Wound Dressings
Source: Adv Healthc Mater. 2026 Aug 17;15(39):e71608. doi: 10.1002/adhm.71608 (PMC13691401; doi:10.1002/adhm.71608)
Supplement: Supplementary file 1 [file ADHM-15-0-s001.docx]

**Supporting Informati****on**

**Rare-Earth–Functionalized Carbon Dot Hydrogels as Mechanically Robust**

**Antibacterial Wound Dressings**

Shih-Han Tsai^#^ ^[a]^, Ramadhass Keerthika Devi^#^ ^[a]^, Muthusankar Ganesan ^[b,c]^, Guo-Ping Chang

Chien ^[b,c,d]^, Chun Che Lin*^[a]^

*^a^Institute of Organic and Polymeric Materials, Research and Development Center for Smart Textile Technology, National Taipei University of Technology, Taipei 106, Taiwan.*

*^b^Super Micro Mass Research and Technology Center, Cheng Shiu University, Kaohsiung 833301, Taiwan.*

^c^*Center for Environmental Toxin and Emerging-Contaminant Research, Cheng Shiu University, Kaohsiung 833301, Taiwan.*

*^d^Institute of Environmental Toxicology and Emerging Contaminants, Cheng Shiu University, Kaohsiung 833301, Taiwan.*

\# Authors equally contributed

Corresponding author: **Chun Che Lin*** ([cclin0530@mail.ntut.edu.tw](mailto:cclin0530@mail.ntut.edu.tw))

Experimental Methods

Materials and Reagents

All chemicals were used as purchased without further purification unless otherwise noted. The chemicals are Polyvinyl alcohol (PVA, Emperor Chemical, FW = 75,000−80,000 g mol^-1^), Dipotassium glycyrrhizinate hydrate (DPG, Tokyo Chemical Industry, ≥ 75%), Sodium tetraborate decahydrate (Borax, Sigma-Aldrich, 100%), Citric acid (CA, Echo Chemical, 99.5%), Urea (Sigma-Aldrich, FW = 60.60 g/mol), and Lanthanum nitrate hexahydrate (La(NO_3_)_3_·6H_2_O, Acros Organics, 99.9%).

**Fabrication of La, N-CDs**

The synthesis procedure of La,N-CDs was adopted from the literature report with slight modifications ^[1]^. La, N-CDs were prepared via the hydrothermal method. First, 2.7 g of citric acid, 0.53 g of urea, and 1.1 g of lanthanum nitrate were added to a beaker, then 50 mL of deionized water was added. The solution was placed on a magnetic stirrer at room temperature for 1 hour, and then the homogeneous solution was transferred into a 100 mL stainless steel reactor with PPL liner for the immediate hydrothermal reaction at 190 °C for 6 hours. After cooling to room temperature, the solution was first passed through a 0.22 μm filter to remove large particles and then transferred to a dialysis bag (MWCO = 1,000 kDa). After dialysis for 12 hours, a light yellow solution of La, N-CDs was obtained. Then, the solution was freeze-dried and left for further characterization.

**Fabrication of La,N-CDs/PBD hydrogels**

First, to prepare the PVA–DPG solution, 10 wt% PVA was dissolved in hot water, and various concentrations of DPG were added to form a transparent solution, which was heated for 1 hours at 95 °C. Subsequently, borax at a concentration of 0.01M was dissolved in ultrapure water to form a borax solution. To prepare the PBD hydrogel, PVA–DPG solution was poured into 2.5 mL of borax solution and heated for 1 h at 95 °C. The mixture was then placed in a mold and frozen at −25 °C for 8 h and subjected to three freeze-thaw cycles. The La,N-CDs/PBD hydrogel was prepared by adding 2 mL of La,N-CDs (2 mg mL^-1^) to PVA–DPG solution, which was then poured into the borax solution and heated for 30 min at 95 °C. The mixture was subsequently placed in a mold, frozen at −25 °C for 8 h, and subjected to three freeze-thaw cycles.

**Characterization**

The Hydrogels were characterized using Fourier transform infrared (FTIR) spectroscopy (PerkinElmer, Spectrum One) in the range of 650-4,000 cm^−1^. Wide-angle X-ray diffraction (XRD; Bruker D8) patterns were recorded in the 10°-70° range at a scanning speed of 10°/min. Small-angle X-ray scattering (SAXS) (NSRRC TLS 23A1) was used to analyze changes in the network structure of hydrogels at different temperatures. The microstructure of the hydrogels was observed using scanning electron microscopy (SEM; Hitachi TM4000Plus). The contact angle analyzer (Phoenix 300) was used to measure the degree of hydrophilicity and hydrophobicity of the hydrogels.

**Moisture content**

According to published procedures, the method for measuring the moisture content of the hydrogel was determined ^[2]^. The weight of hydrogel was measured before and after drying in the oven. The water content of hydrogel was calculated using the following formula (1):

$\mathrm{Moisturecontent}\left( \% \right) = \left[ \frac{\left( W_{w} - W_{d} \right)}{W_{w}} \right]\times100\%$	(1)

where W_w_ is the weight of the wet hydrogel and W_d_ is the weight of the hydrogel after drying. Each sample was measured three times, and the average value was used.

**Swelling ratio**

According to published procedures, the method for measuring the swelling ratio of the hydrogel was determined ^[3]^. The dried hydrogels were weighed before being immersed in phosphate buffer solution (PBS) with pH values of 5.0 and 7.4. The hydrogels were removed at different time intervals. After removing excess surface water with filter paper, the weight of the swollen hydrogels was measured. The swelling ratio was calculated using equation (2):

$Swelling ratio \left( \% \right) = \left[ \frac{\left( W_{s} - W_{d} \right)}{W_{d}} \right]\times100$%	(2)

where W_s_ is the weight of the hydrogel after swelling equilibrium and W_d_ is the initial weight of the dry hydrogel. Each sample was measured three times, and the average value was used.

**Release rate**

The test method first requires establishing a standard curve for N-CDs and La,N-CDs by measuring the absorption values at different concentrations using absorption spectra ^[4]^. The absorption values at the absorption peak and the corresponding concentrations were linearly fitted to establish a standard curve and linear equation. The hydrogels were soaked in 100 mL of PBS at pH values of 5.0 and 7.4, then placed on a shaker at 70 rpm and 37 °C. At different time points, 3 mL of solution was aspirated, and its absorption value was measured. Subsequently, 3 mL of fresh PBS was added to the mixture, maintaining a constant total volume. Each set of experiments was repeated 6 times. Based on the measured absorption values, the concentration of N-CDs and La, N-CDs released at different time points, was calculated using the standard curve equation. Finally, the total release amount was calculated according to equation (3):

$Carbon dots Released \left( \% \right) = \frac{(Carbon dots released)}{(Carbon dots loaded)}\times100$%	(3)

**Hemolysis test**

Centrifuge mouse blood at 8,000 rpm for 10 min. The resulting red blood cells were washed three times with PBS and then resuspended in PBS to obtain a 5% (v/v) suspension. Next, 1 mL of the red blood cell suspension was mixed with the hydrogel sample and shaken at 70 rpm for 1 h in a 37 °C incubator ^[5]^. PBS buffer was used as the negative control, while a 0.1% Triton X-100 solution was used as the positive control. After the reaction, centrifuge the sample again at 8,000 rpm for 10 min. Transfer the supernatant to a 96-well microplate, measure the absorbance value at 540 nm, and calculate the hemolysis rate according to equation (4):

$Hemolysis ratio \left( \% \right)=\left[ \frac{\left( A_{s} - A_{b} \right)}{(A_{p} - A_{b})} \right]\times100$%	(4)

Where A_s_ is the absorbance value of the hydrogel, A_b_ is the absorbance value of the PBS solution, and A_p_ is the absorbance value of the Triton X-100 solution.

**Cytotoxicity test**

To assess the cytotoxicity of hydrogels, a $0.5 \mathrm{cm}\times0.3 \mathrm{cm}$ piece of hydrogel was placed in a 48-well plate, and L929 cells were seeded on to the hydrogel at a density of 5 × 10^4^ cells and allowed to adhere for 24 h. The biocompatibility of hydrogels was evaluated using the CCK-8 assay. The control group consisted of cells cultured in a medium without hydrogels.

**Antibacterial test**

The antibacterial properties of the hydrogel were studied using gram-negative bacteria (*E. coli*) and gram-positive bacteria (*S. aureus*) as model organisms. The bacteria were added to 4 mL of Luria–Bertani nutrient broth and cultured for 16 h at 37 °C. To determine the cell density, the absorbance of the bacterial solution was adjusted to between 0.4 and 0.6 using a UV–vis spectrophotometer at a wavelength of 600 nm. The bacterial solution (50 μL) was then poured on the agar plate and spread evenly, after which the sample was added. The inhibition zone was observed after 24 h.

**Animal Analysis**

These mice were purchased from BioLASCO Taiwan Co., Ltd. (Yilan City, Taiwan) and acclimated for at least one week before the experiments. Standard food and water were provided ad libitum. All animal procedures were approved by the Laboratory Animal Center of Taipei Medical University under approval number LAC-2022-0203. The rats were anesthetized by injection, and two full-thickness circular skin wounds with a diameter of 1.5 cm were created on the shaved dorsal skin of each rat. This time point was recorded as day 0. The rats were randomly divided into four groups, with three rats in each group (n = 3): control, PBD hydrogel, N-CDs/PBD hydrogel, and La,N-CDs/PBD hydrogel. The control group did not receive hydrogel treatment. After hydrogel application, the wound area was covered with artificial skin and sutured around the wound to prevent displacement of the hydrogel dressing. Wound photographs were taken from the same position on days 3, 7, 10, and 14. Wound areas were measured using ImageJ software, and the wound-healing rate was calculated according to Eq. (5):

$Wound healing rate \left( \% \right)=\frac{\mathrm{Area}\left( Day 0 \right) -Area(Day x)}{Area(Day 0)}\times100$%	(5)

where Area day 0 is the initial wound area and Area day x is the wound area at the selected time point. In addition, wound tissues collected on days 7 and 14 were analyzed by H&E and Masson staining.

**Statistical Analysis**

All data were analyzed and visualized by Origin 2019 software (USA). Quantified data are presented as mean ± standard deviation (SD) with the sample size of n = 3.

**Figure S1.** HR-TEM image of N-CDs.

**Figure S2.** High-resolution XPS spectra of (a) C1s, (b) N1s and (c) O1s of N-CDs; and (d) C1s, (e) N1s and (f) O1s of La,N-CDs.

**Figure S3**. EDS elemental analysis of (a) N-CDs and (b) La,N-CDs.

**Figure S4.** (a) Absorption and fluorescence spectra of N-CDs, (b) Fluorescence spectra of N-CDs under various excitation wavelengths, (c) Absorption and fluorescence spectra of La,N-CDs, (d) Fluorescence spectra of La,N-CDs under different excitation wavelengths. The illustrations are photos of N-CD and La,N-CD taken under daylight and laser light.

**Figure S5.** Dose-dependent cytocompatibility of La,N-CDs. (a) Representative L929 cell images before and after treatment with La,N-CDs (10–0.1%), with (b) corresponding cell viability analysis. High concentrations (10–1%) induce marked cytotoxicity, whereas 0.1% maintains viability comparable to the negative control (NC). Error bars indicate mean ± SD.

**Figure S6**. (a) Mechanical properties of hydrogels at different concentrations of DPG. (b) XRD and (c) FTIR spectra of PVA, borax, DPG, PVA/borax hydrogel, and PVA/borax/DPG hydrogel. (d) FTIR spectra of hydrogels synthesized with different concentrations of DPG.

**Figure S7.** SEM images of (a-c) PB, (d-f) PBD, (g-i) N-CDs/PBD, and (j-l) La,N-CDs/PBD hydrogels. Scale bars are 40 μm, 30 μm, and 20 μm.

**Figure S8.** (a) Contact angle and (b) moisture content analysis of PB, PBD, N-CDs/PBD and La,N-CDs/PBD hydrogels.

**Figure S9.** Swelling rate of N-CDs/PBD and La,N-CDs/PBD hydrogels in PBS at pH 5.0 and 7.4.

**Figure S10.** Representative agar plate images from the CFU assay of E. coli and S. aureus after contact with the La,N-CDs/PBD hydrogel under the tested conditions.

**Figure S11**. H&E staining analysis of PBD, N-CDs/PBD, and La,N-CDs/PBD hydrogels. Scale bars are 100 μm and 50 μm.

**Table S1. Comparison of La,N-CDs/PBD with Representative Antibacterial Hydrogel Wound-Dressing Systems.**

| **Hydrogel system** | **Antibacterial component/strategy** | **Antibacterial**  **evaluation** | **Wound model/healing outcome** | **Biocompatibility/safety** | **Key comparison** | **Ref.** |
| --- | --- | --- | --- | --- | --- | --- |
| La,N-CDs/PBD hydrogel | La-functionalized N-CDs embedded in DPG-reinforced PVA-borax hydrogel | Inhibition zones against E. coli and S. aureus; CFU plate assay; SEM bacterial morphology | Full-thickness rat wound model; 99.1 +/- 1.9% wound closure at day 14 | L929 viability >80%; hemolysis <5%; ISO 10993-5 agar diffusion cytotoxicity grade 0 | Non-antibiotic rare-earth/carbon-dot antibacterial interface with high wound closure and acceptable biological safety | This work |
| Cationic carbon-dot-releasing hydrogel | Cationic CDs released from pectin/acrylic acid hydrogel network in response to bacteria-associated environmental changes | Antibacterial activity against bacteria; fluorescence performance | Proposed as a candidate wound-healing hydrogel system | Reported high biocompatibility | CD-based antibacterial hydrogel reference system; relies on cationic CD release | ^[6]^ |
| Injectable self-healing D/epsilon-polylysine hydrogel | Antibacterial CDs conjugated with epsilon-polylysine to form an injectable self-healing hydrogel | Broad antibacterial evaluation for bacteria-infected wound treatment | Promoted bacteria-infected wound healing with epithelialization and angiogenesis | Reported satisfactory biocompatibility | CD/polycation-based antibacterial self-healing hydrogel for infected wound repair | ^[7]^ |
| Conductive self-healing hydrogel with dual dynamic bonds | OSD/CMC/Fe hydrogel combined with polydopamine-encapsulated poly(thiophene-3-acetic acid); NIR-assisted photothermal antibacterial function | High antibacterial efficiency; photothermal antibacterial testing | Infected full-thickness mouse wound model; wound size reduced to ~12% after 14 days for the optimized hydrogel + NIR group | Reported good biocompatibility, hemostasis, antioxidant activity, conductivity, and self-healing | Advanced infected-wound hydrogel with conductivity and photothermal activity; requires NIR irradiation for the strongest antibacterial effect | ^[8]^ |
| Intrinsic antibiofilm and antioxidative hydrogel dressing | PEG hydrogel tethered with cationic polyimidazolium and N-acetylcysteine | MDR bacterial biofilm antibacterial/antibiofilm evaluation | Biofilm-infected diabetic murine wound model; accelerated wound closure | Low-leaching, antibiotic-free, metal-free, and nanoparticle-free design | State-of-the-art intrinsic antibacterial/antioxidative hydrogel; strong infected diabetic wound benchmark | ^[9]^ |

**Reference**

[1]	H. Qi, L. Qiu, X. Zhang, T. Yi, J. Jing, R. Sami, S. F.Alanazi, Z. Alqahtani, M. D. Aljabri, M. M. Rahman, *RSC Adv.* **2023**, *13*, 2663.

[2]	M. Alboofetileh, M. Rezaei, H. Hosseini, M. Abdollahi, *J. Food Eng.* **2013**, *117*, 26.

[3]	T. Cui, Y. Wu, Z. Wang, Q. Ban, J.Cheng, *Int. J. Biol. Macromol.* **2024**, *254*, 127929.

[4]	Q. Duan, Y. Ma, M. Che, B. Zhang, Y. Zhang, Y. Li, W. Zhang, S. Sang, *J. Drug Deliv. Sci. Technol.* **2019**, *49*, 527.

[5]	Y. Jiang, G. Li, J. Qiao, P. Yan, K. Tang, *Colloids Surfaces B Biointerfaces* **2023**, *228*, 113424.

[6]	F. Cui, J. Sun, J. Ji, X. Yang, K. Wei, H. Xu, Q. Gu, Y. Zhang, X. Sun, *J. Hazard. Mater.* **2021**, *406*, 124330.

[7]	C. Mou, X. Wang, J. Teng, Z. Xie, M. Zheng, *J. Nanobiotechnology* **2022**, *20*, 368.

[8]	L. Qiao, Y. Liang, J. Chen, Y. Huang, S. A. Alsareii, A. M. Alamri, F. A. Harraz, B. Guo, *Bioact. Mater.* **2023**, *30*, 129.

[9]	D. Pranantyo, C. K. Yeo, Y. Wu, C. Fan, X. Xu, Y. S. Yip, M. I. G. Vos, S. H. Mahadevegowda, P. L. K. Lim, L. Yang, *Nat. Commun.* **2024**, *15*, 954.
